# Postpartum depression symptoms in survey-based research: a structural equation analysis

**DOI:** 10.1186/s12889-020-09999-2

**Published:** 2021-01-27

**Authors:** Che Wan Jasimah Bt Wan Mohamed Radzi, Hashem Salarzadeh Jenatabadi, Nadia Samsudin

**Affiliations:** Department of Science and Technology Studies, Faculty of Science, University of Malaya, 50603 Kuala Lumpur, Malaysia

**Keywords:** Postpartum depression symptoms, Structural equation modeling, Obesity, Edinburgh postnatal depression scale

## Abstract

**Background:**

Since the last decade, postpartum depression (PPD) has been recognized as a significant public health problem, and several factors have been linked to PPD. Mothers at risk are rarely undetected and underdiagnosed. Our study aims to determine the factors leading to symptoms of depression using Structural Equation Modeling (SEM) analysis. In this research, we introduced a new framework for postpartum depression modeling for women.

**Methods:**

We structured the model of this research to take into consideration the Malaysian culture in particular. A total of 387 postpartum women have completed the questionnaire. The symptoms of postpartum depression were examined using the Edinburgh Postnatal Depression Scale (EPDS), and they act as a dependent variable in this research model.

**Results:**

Four hundred fifty mothers were invited to participate in this research. 86% of the total distributed questionnaire received feedback. The majority of 79.6% of respondents were having depression symptoms. The highest coefficients of factor loading analysis obtained in every latent variable indicator were income (β = 0.77), screen time (β = 0.83), chips (β = 0.85), and anxiety (β = 0.88). Lifestyle, unhealthy food, and BMI variables were directly affected by the dependent variable. Based on the output, respondents with a high level of depression symptoms tended to consume more unhealthy food and had a high level of body mass indexes (BMI). The highest significant impact on depression level among postpartum women was unhealthy food consumption. Based on our model, the findings indicated that 76% of the variances stemmed from a variety of factors: socio-demographics, lifestyle, healthy food, unhealthy food, and BMI. The strength of the exogenous and endogenous variables in this research framework is strong.

**Conclusion:**

The prevalence of postpartum women with depression symptoms in this study is considerably high. It is, therefore, imperative that postpartum women seek medical help to prevent postpartum depressive symptoms from worsening.

## Background

The number of people diagnosed with depression has been steadily increasing over the years. It affects the patient’s work performance, financial status, and interpersonal relationships [1]. Depression can be observed from the individual’s passive behavior such as loss of interest, feelings of guilt, low self-respect, sleep-deprived, poor appetite, constantly being unhappy, or showing signs of fatigue [2, 3]. Living with depression causes a serious disability to the patient because it is associated with mental and behavioral disorders [4]. It is highly probable that this condition affects the patient’s physical well-being leading to increased morbidity and mortality [1, 5, 6]. In 2017, the World Health Organization (WHO) reported that over 300 million people suffered from depression [7]. However, previous studies showed that depression typically occurred among women, as opposed to men [8]. The primary reasons for depression among women were attributed to hormonal transition, such as puberty, pregnancy, and menopausal changes [1]. In particular, after giving birth, a woman needs extra care and should be given the right kind of health care priorities. Moreover, any unpleasant act can cause depression at this stage which will be devastating for the whole family [9]. Postpartum depression (PPD) was identified as the number one complication that plagued one in seven women [10]. It has been estimated that more than 20% of women globally suffer from PPD [11]. PPD usually occurs 6 to 8 weeks after childbirth, which may lead to a decrease in an individual’s daily performance [12]. Mothers are commonly faced with discomfort due to physical changes, poor sleeping quality, and various uncertainties related to their newborns in the postpartum stage [13].

Today, PPD has become a major worldwide health problem. Even so, many women with this mental illness were not medically diagnosed [12]. Several factors associated with PPD have been identified, although the specific causes remained unknown [11]. Previous studies have shown that depression and obesity were closely linked [14, 15]. The risk of depression was increased by almost 37% due to obesity among women. It is quite common among women to gain excessive weight during pregnancy and the postpartum period [16]. Ertel et al. [17] and LaCoursiere et al. [18] also claimed that there was some evidence regarding pre-pregnancy obesity, which may lead to PPD. This claim was also supported by similar research conducted by Ruyak et al. [19]. Mgonja and Schoening [10] and Ezzeddin et al. [20] further placed emphasis on the issue by examining factors, aside from obesity, that could lead to the development of PPD; the factor included poor marital relationships, divorce, substance abuse, violence, other mental health diagnoses, low educational levels, unwanted or unexpected pregnancies, complicated labor, and a weak health care support system. This assertion by Mgonja and Schoening were reinforced by similar findings by Azale et al. [21], Zhao et al. [22], and Ukatu, Clare, and Brulja [11] who focused on factors leading to maternal depression. Hence, Bledsoe et al. [23] concluded that the negative outcomes from social, educational, health, and economic aspects tend to contribute a high possibility for the development of PPD among women. There is significant evidence that genetics and biochemical factors (brain chemistry), personality style, illness, and significant transitions in life, including adjusting to living with a new baby, may also contribute to PPD [24, 25]. Postpartum depression has also been linked to women’s lifestyle choices, such as sleep quality [26], exercise [27], and prenatal smoking [28]. Dos Santos et al. [29] concluded that women who were diagnosed with maternal depression also experienced a higher risk of eating disorders during their pregnancies. Unhealthy eating habits developed among pregnant women because they were afraid to gain weight whenever they ate. Nevertheless, pregnant women with an eating disorder could have healthier food options, and some were concerned with their body shape rather than their body weight [30]. In other words, body dissatisfaction seemed to be a predictor of weight gain during pregnancy due to lifestyle factors (e.g., physical activity, diet, stress, and fatigue levels) [31]. In essence, a mother needs to have healthy food in order to supply the right kinds of nutrition to her unborn child [32].

Unfortunately, there were very few studies that investigated the impact of lifestyle and food intake by considering the body mass index (BMI), which may be associated with the PPD occurrence among women. Previous studies on PPD were infrequent; some utilized a modeling technique to measure the output and estimated the suggested indicators. Despite the contribution of these variables to PPD, a combined analysis of indicators involved in postpartum depression is surprisingly non-existent. A Structural Equation Modelling (SEM) analysis would allow the integration of variables such as demographic, lifestyle, and food intake in a conceptual model, which interrelates each of these variables to PPD. Therefore, in this research work, the authors aimed to analyze the factors, which contribute to PPD, and its relationship with socio-demographics, the lifestyle of postpartum women, healthy food intake, unhealthy food intake, and BMI range, which affects PPD by using SEM analysis.

## Methods

### Research framework

**Fig. 1:**
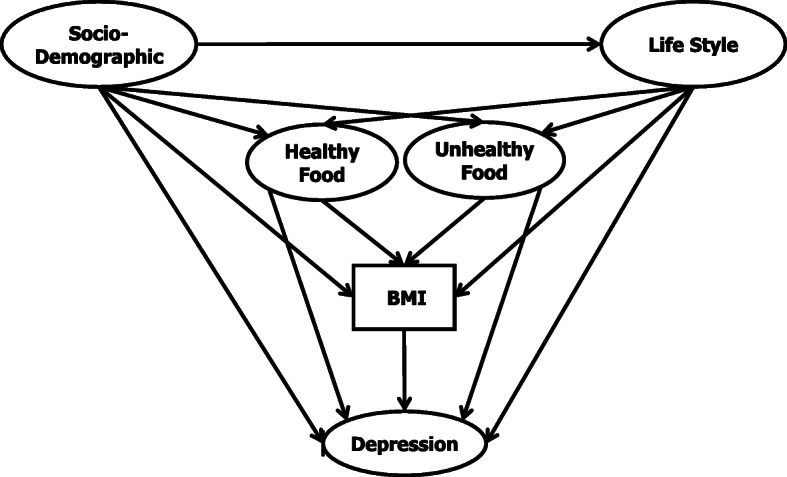
Research framework

The authors designed a research framework that correlated to PPD, as shown in Fig. 1. The conceptual framework of the research model includes an integrated model capable of providing an inclusive evaluation of the latent and observed variables within the SEM framework. The framework comprises socio-demographics as the initial independent variable and the depression level as the dependent variable. The remaining variables which acted as mediators were lifestyle, healthy food, unhealthy food, and BMI. As the BMI needed to be calculated based on the respondent’s weight and height, it was the only measured variable in our research framework. These variables have been taken into consideration the Malaysian culture because Malaysia is a multiracial country and have various ethnic groups [33]. Thus, we had chosen the variables wisely which were practical among Malaysian mothers.

The research framework provides a clear view of the study is carried out. By constructing the framework, it will lead this research to achieve its objective. This research framework was constructed using a combination of a theoretical framework with the addition of some new ideas to analyze the model. According to previous studies, socio-demographic variables played a significant role in establishing the relationship between the variables to postpartum women. These variables include with their lifestyles [34], healthy food intake [35], unhealthy food intake [36], BMI [37], and depression [38]. Lifestyle intervention during the postpartum period that give an impact on healthy food and unhealthy food intake [39], BMI [40], and depression [41] had also been investigated by other researchers.

Apart from that, food consumption among postpartum women has an interrelationship with BMI that claimed by research conducted by Kay et al. [42]. It was reported by Nathanson et al. [35] that healthy food intake closely associated with depression. Yet, Faria-Schutzer et al. [43] claimed that unhealthy food intake correlated with depression. Based on Ertel et al.’s [17] research, the postpartum BMI level can be affected by the depression level.

### Materials and measurements

In this research, socio-demographics were measured as the initial independent variable, which includes four indicators, i.e., age group, educational background, working experience, and income household per month. The age range was classified into four groups: 21 to 25 years old, 26 to 30 years old, 31 to 35 years old, and over 35 years old. The educational background of the respondents was categorized as “Less than high school”, “High school”, “Diploma”, “Bachelor’s degree” and “Master’s degree or Ph.D.”. The respondents were asked about their working experience, which was categorized as “no job experience”, “1 to 3 years”, “4 to 6 years”, “7 to 10 years”, and “more than 10 years”. The last question in the socio-demographic section was based on to the monthly household income in Ringgit Malaysia (RM) and the responses were classified as “Less than RM 2,000”, “RM 2,000-RM 3,000”, “More than RM 3,000 to RM 4,000”, “More than RM 4,000 to RM 5,000”, and “Over RM 5,000”.

Apart from that,the authors measured lifestyle based on Nakayama, Yamaguchi’s study [44] in which the authors selected a few indicators such as the average working hours per day, physical activity per week, and average sleeping hours per day. Besides, daily screen time (e.g., TV, smartphone, tablet, etc.) was added to measure the lifestyle of the respondents in terms of social media, which corresponded to Khajeheian et al.’s research [45]. Regarding the average working hours per day, the responses consisted of five categories denoted by “none”, “less than 7 hours”, “7 to 8 hours”, “8 to 9 hours” and “more than 9 hours”. As for the frequency of physical activity per week, this was indicated as “none”, “1 time”, “2 times”, “3 times”, “4 times”, and “more than 4 times”. The average screen time per day was denoted as “less than 1 hour”, “1 to 2 hours”, “2 to 3 hours”, “3 to 4 hours”, and “more than 4 hours”. The average sleeping hours per day were indicated as “less than 6 hours”, “6 to 7 hours”, “7 to 8 h”, “8 to 9 h”, and “more than 9 h”.

In addition to the mediators, the authors considered healthy and unhealthy food separately in this study. Fruits, vegetables, and whole grains were selected as ‘healthy food’ variables, whilst fast food such as sweets, chips, and soft drinks was categorized as ‘unhealthy food’ [42, 46]. The respondents were asked about their healthy and unhealthy food intake, and the responses were based on a five-point scale (“never”, “rarely”, “sometimes”, “mostly”, and “always”) which have been used in the previous studies [47].

WHO defined BMI as a simple index of weight-to-height of an individual and calculated according to the formula, BMI = ((weight in kilograms)/ (height in meters)^2^) [48]. There are four categories of BMI based on the BMI range including “underweight for less than 18.5 kg/m^2^”, “normal for ranges between 18.5 to less than 25.0 kg/m^2^”, “overweight for ranges between 25.0 kg/m^2^ to less than 30.0 kg/m^2^” and “obese for ranges between 30.0 kg/m^2^ and above” [49].

For the dependent variable, the authors measured the depressive symptoms using the Edinburgh Postnatal Depression Scale (EPDS) questionnaire to validate prenatal and postpartum occurrences [50–53]. Moreover, previous studies have shown that EPDS had been validated in Malaysian samples as well [54, 55]. EPDS was calculated using a four-point scale (0–4) for each item to measure the frequency of the depressive symptoms developed in the postpartum period. A total of 10 items was used in the EPDS to estimate the depressive symptoms of respondents that needed to be answered. The total score for the EPDS questions was then grouped into four categories with a different interpretation. A 0–9 score was categorized as “normal”, scores of 10–11 were categorized as “slightly increased risk”, scores of 12 to 15 as “increased risk” and those more than 15 were listed as “likely depression” [56].

### Structural equation modeling (SEM)

The SEM technique was chosen to be used in this research as it was recognized as a suitable method that would most likely help a researcher to understand better the latent variables concepts and the interactions within the model. Several previous studies had used the structural equation methodology [57, 58] in their studies due to its features. The features of SEM technique include being:

- Capable of estimating and examining the direct and indirect interrelationships which exist among the variables in the research study [59].
- Capable of showing the relationship among dependent variables, which helps indicate the simultaneous estimation of more than one exogenous and endogenous variable [60].

### Sampling

For sampling, we used a cross-sectional analysis. The survey data was collected from each subject at one point in time. Based on Hair et al. [61], the required sample size depended on the number of latent variables in the study, including the number of indicators. In other words, a)100 respondents were needed due to five or less latent variables, of which each of the latent variables included at least three indicators, b) 150 respondents were needed due to seven or less latent variables, of which each of the latent variables included at least three indicators, c) 300 respondents were needed since seven or less latent variables existed, of which some of the latent variables had less than three indicators, d) 500 respondents were needed due to the existence of more than seven latent variables, of which some of the latent variables had less than three indicators. In this research framework, the authors had five latent variables, to be precise. Thus, the authors were required to consider at least 150 respondents for a suitable sample size.

The respondents were selected randomly using proportionate stratified random sampling and the data were collected for almost 6 months. The questionnaires were self-administered and have been distributed online, by sending respondents the link. However, for respondents who don’t have access to the internet, they were given the printed questionnaire to fill up. The authors distributed 450 questionnaires to postpartum women who were living in Kuala Lumpur, the most highly populated city in Malaysia. We excluded the women who are not living in Kuala Lumpur from the analysis. A total of 387 completed questionnaires were received from the respondents. The data were collected from nine maternal and child health clinics around Kuala Lumpur. The maternal and child health clinics that we went for data collection were in the neighborhood area which most of the patients are living nearby the clinics. We chose to collect the data at the maternal and child health clinics because it was easy to recognize mothers in their one-year postpartum period as mothers went to the clinics for medical check-ups. The respondents were selected randomly as long as they met the main criteria, i.e., in the first postpartum year of their latest pregnancy. The survey was conducted under the backingsof the University of Malaya’s Research Ethics Committee approval (UM.TNC2/RC/H&E/UMREC 127) and with the grant obtained from the University of Malaya (Grant No.: GPF066B-2018andGC002C-17HNE).

## Results

Table 1 shows the descriptive statistics of this research. The respondents are made up of Malays (43.7%), Chinese (34.9%), and Indians (21.4%), who were mostly around 31 years of age and older. The majority of participants were educated and gained an income of over RM 3000 per month with 1 to 10 years of working experience. Based on the weight and height provided by the respondents, 38.0% of participants were obese, 28.7% were overweight, 24.8% were normal, and 8.5% were underweight. Regarding lifestyle, only 26.1% of respondents did not take part in any physical activities. The average sleeping hours of the respondents were around 7 to 9 h, coupled with 8 to 9 h working day. The mean screen time hours recorded were 4.08 (SD: 0.85 h) per day. In terms of the food intake among postpartum women, the majority of respondents mostly consumed fruits, vegetables, whole grains, fast food, and sweets. Apart from that, a large number of respondents always consumed chips and soft drinks. Based on the calculated EPDS score, only 20.4% of the respondents were normal. Depression levels for the rest of respondents were 25.3% (slightly increased risk), 32.6% (increased risk), and 21.7% (likely depression).

**Table 1:** Descriptive statistics

Number Percentage					
**BMI** (kg/m^2^)					
Underweight (n, %)				33	8.5%
Normal (n, %)				96	24.8%
Overweight (n, %)				111	28.7%
Obese (n, %)				147	38.0%
**Age** (n, %)					
21 to 25 years old				25	6.5%
26 to 30 years old				89	23.0%
31 to 35 years old				156	40.3%
Over 35 years old				117	30.2%
**Education** (n, %)					
Less than high school				69	17.8%
High school				44	11.4%
Diploma				102	26.4%
Bachelor				137	35.4%
Master or PhD				35	9.0%
**Income** (n, %)					
Less than RM 2000				33	8.5%
RM 2000-RM 3000				42	10.9%
More than RM 3000- RM 4000				135	34.9%
More than RM 4000- RM 5000				141	36.4%
Over RM 5000				36	9.3%
**Job Experience** (n, %)					
No job experienced				33	8.5%
1–3 years				75	19.4%
4–6 years				95	24.5%
7–10 years				113	29.2%
More than 10 years				71	18.3%
**Physical Activity** (n, %)					
None				101	26.1%
1 time				75	19.4%
2 times				96	24.8%
3 times				44	11.4%
4 times				42	10.9%
More than 4 times				29	7.5%
**Screen Time** (n, %)					
Less than 1 h				0	0.0%
1–2 h				0	0.0%
2–3 h				126	32.6%
3–4 h				105	27.1%
More than 4 h				156	40.3%
**Sleeping** (n, %)					
Less than 6 h				24	6.2%
6–7 h				46	11.9%
7–8 h				159	41.1%
8–9 h				122	31.5%
More than 9 h				36	9.3%
**Working** (n, %)					
None				36	9.3%
Less than 7 h				24	6.2%
7–8 h				73	18.9%
8–9 h				221	57.1%
More than 9 h				33	8.5%
Number (%)	Never	Rarely	Sometimes	Mostly	Always
**Healthy Food**					
Fruits	0 (0.0%)	35 (9.0%)	124 (32.0%)	172 (44.4%)	56 (14.5%)
Vegetables	0 (0.0%)	21 (5.4%)	133 (34.4%)	144 (37.2%)	89 (23.0%)
Whole grains	0 (0.0%)	32 (8.3%)	113 (29.2%)	151 (39.0%)	91 (23.5%)
**Unhealthy Food**					
Fast food	0 (0.0%)	0 (0.0%)	26 (6.7%)	192 (49.6%)	169 (43.7%)
Sweets	0 (0.0%)	0 (0.0%)	37 (9.6%)	186 (48.1%)	164 (42.4%)
Chips	0 (0.0%)	0 (0.0%)	44 (11.4%)	162 (41.9%)	181 (46.8%)
Soft drinks	0 (0.0%)	0 (0.0%)	65 (16.8%)	151 (39.0%)	171 (44.2%)
**Depression Level (n, %)**					
Normal			79 (20.4%)		
Slightly increased risk			98 (25.3%)		
Increased risk			126 (32.6%)		
Likely depression			84 (21.7%)		

Fornell and Larcker [62] claimed that the validity and reliability of a survey needed to fit the requirements of the SEM analysis. The validity is supposed to be tested based on the Cronbach’s alpha coefficient. Every latent variable in the research framework should be equal to or higher than 0.7. The Cronbach’s alpha value in this research was more than 0.7, which aligned with the conditions required to validate this research. To examine the reliability of the research work, a loading factor higher than 0.7 needed to be obtained for the latent variable indicator (see Table 2). In Table 2, several indicators obtain a factor loading coefficient of less than 0.7, which means that these indicators need to be eliminated from the SEM analysis.

**Table 2:** Factor loading analysis

Socio-demographics	
Age ^a^	0.63
Education	0.73
Income	0.77
Job Experience	0.72
Lifestyle	
Physical Activity	0.74
Screen Time	0.83
Sleeping	0.72
Working	0.79
Healthy and Unhealthy food	
Fruits ^a^	0.65
Vegetables	0.76
Whole grains	0.81
Fast food	0.73
Sweet	0.76
Chips	0.85
Soft drinks	0.83
Depression	
I have been able to laugh and see the funny side of things (Q1)	0.72
I have looked forward with enjoyment to things (Q2)	0.76
I have blamed myself unnecessarily when things went wrong (Q3) ^a^	0.62
I have been anxious or worried for no good reason (Q4)	0.88
I have felt scared or panicky for no very good reason (Q5) ^a^	0.54
Things have been getting on top of me (Q6)	0.81
I have been so unhappy that I have had difficulty sleeping (Q7)	0.73
I have felt sad or miserable (Q8) ^a^	0.68
I have been so unhappy that I have been crying (Q9)	0.79
The thought of harming myself has occurred to me (Q10)	0.74

^a^obtained factor loading less than 0.70 and should be eliminated from SEM analysis

**Fig. 2:**
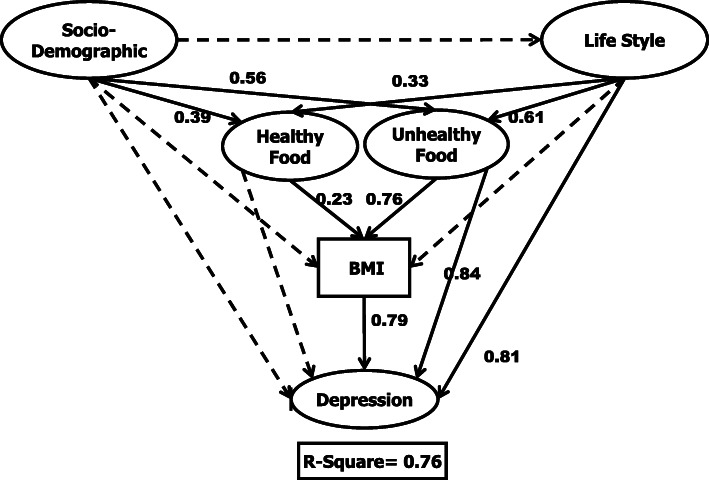
Final output of structural model

The reliability of the research also needed to be fitted with another test after the elimination of these unfit indicators. All latent variables should obtain an equal or higher coefficient than 0.5 of the average variances extracted (AVE). AVE analysis for latent variables in this research achieved more than 0.5. Thus, in this research work, the validity and reliability features are fulfilled. The suitability of the research model was tested using the model fitting analysis. The comparative fit index (CFI), normed fit index (NFI), relative fit index (RFI), incremental fit index (IFI), the goodness of fit index (GFI), and Tucker-Lewis index (TLI) coefficient of this research were above 0.9, which means that the research data was acceptable. The structural model in the SEM analysis helped to recognize the connection between research variables and the considered conceptual model. Figure 2 shows the output of the structural model for postpartum women. From the pre-established 14 relationships between the research variables, only five relationships, represented by the dashed black arrow, were deemed not significant.

Figure 2 presents that R-square is equal to 0.76. which means that 76% of depression variations depend on BMI, healthy food intake, unhealthy food intake, lifestyle, and socio-demographics among postpartum women. The rest, 24% of depression variation belongs to other variables that were not involved inside the model. Moreover, from 14 relationships among research variables, nine of them have significant relationships. Among the four latent variables and one measurement variable, two of the latent variables i.e. unhealthy food and lifestyle, have a significant relationship with depression. Additionally, BMI as the only measurement variable has a significant relationship with depression. The highest relationships belong to unhealthy food intake → depression (0.84), lifestyle → depression (0.81), and BMI → depression (0.79). Socio-demographics, as the main independent variable has a significant relationship with healthy and unhealthy food intakes and there is no significant relationship with the lifestyle, BMI, and depression. However, socio-demographics has an indirect effect on depression through food intake mediators (socio-demographics →unhealthy food intake → BMI → depression) and (socio-demographics →unhealthy food intake → BMI → depression). As a result, socio-demographics has no direct effect on depression but have an indirect significant effect. Besides, socio-demographics also do not have a significant direct effect on BMI. It means that postpartum women with any spectrum of socio-demographics including age, education, income, and job experience has no significant effect on their BMI and depression. The correlation of these indicators as a latent variable indicates that their BMI and depression will be significantly affected through their food intake. Meanwhile, Table 3 presents the *p*-value of the final output obtained was significant (approximately *p*-value < 0.05).

**Table 3:** *P*-value output

Relationships between variables	Significant
Socio-demographics →Healthy food	< 0.0104
Socio-demographics → Unhealthy food	< 0.0094
Lifestyle → Healthy food	< 0.0177
Lifestyle → Unhealthy food	< 0.0069
Lifestyle → Depression	< 0.0010
Healthy food → BMI	< 0.0241
Unhealthy food → BMI	< 0.0010
Unhealthy food → Depression	< 0.0010
BMI → Depression	< 0.0010

## Discussion

This paper aimed to introduce a new postpartum depression model, which is designed based on factors associated with depression symptoms using the SEM technique. The depression levels of postpartum women were set as the dependent variable, and socio-demographics were maintained as an independent variable. In this research framework, lifestyle, healthy food, unhealthy food, and BMI acted as mediators. Based on previous theories and frameworks of postpartum depression, the authors designed an improved study model, as shown in Fig. 1. The authors succeeded in gathering the questionnaires from 387 women diagnosed with postpartum. The respondents were into their first postnatal year, which matched the previous research conducted by Kubota et al. [51].

For this research model, 14 out of nine relationships among the variables were significant, with a positive coefficient. In Fig. 2, we simplified our research model output. Thus, the significant impact of socio-demographics on healthy and unhealthy food is 0.39 and 0.56, respectively. It can be interpreted that respondents who have more money, good education backgrounds, and longer work experience tend to consume more unhealthy food than healthy food. Previous research [63] has reported that working mothers tend to feed the family with fast-food as it is the easiest and fastest way to prepare the meal. Yet, some research also mentioned that working mothers had bettereating practices [64]. These show a very contradictory output from the prior studies. On the other hand, it is claimed by Zagorsky and Smith [65] that adults from different levels of socio-demographicspreferred to consume fast food. This claim is supported by similar research done by Fryar, Hughes, Herrick, Ahluwalia [66]. Good educational background was linked with a greater frequency of fast food consumption among women as well [67]. In this research, we obtained a result showing that a high level of socio-demographics chose to eat unhealthy food more. It is proven before that the rationale for people consuming fast food due to convenience and wanted to socialize [68].

The age group indicator was eliminated from the SEM analysis, as the coefficient of factor loading did not achieve the required standard value. Additionally, the lifestyle variable is significant in terms of affecting the dependent variable in this research model. Referring to the factor loading analysis in Table 2, the lifestyle indicators show that the average screen time hour has the highest loading factor, followed by the average working hour indicator. Previous studies have shown a positive correlation between smartphone addiction and depression [69]. The lifestyle factor had a significant impact on both food intake categories. An increase in terms of lifestyle will promote an increase in depression levels and food consumption, in particular, unhealthy food. Berk et al. [70] summarized that poor lifestyle and unhealthy diet contributed to depression.

Apart from that, healthy and unhealthy foods show a significant correlation with BMI in the structural model. In previous studies, it was reported that food consumption contributed to the BMI range [70, 71]. To be exact, the quantity of the food that we consumed affected the BMI level. This will occur when you are eating healthy food but in a substantial amount, which will consequently lead to an increase in the BMI level. So, to apply healthy eating behavior, it is better to know the number of calories needed for the individual’s body. Based on the factor loading analysis in Table 2, the chips indicator had the highest coefficient among the indicators of other food categories. Previous studies also claimed that snacks (i.e., chips) have an impact on the BMI of postpartum women [71]. When a comparison was made between the food categories’ impact on BMI, unhealthy food has a higher significant coefficient than healthy food. Therefore, an increase in unhealthy food intake will also increase the BMI levels of respondents. In Malaysia, unhealthy foods are easy to find and mostly cheaper than healthy food. As in Kuala Lumpur, a lot of 24-h restaurants are available, especially fast-food premises [72]. Thus, with the availability of easy food at any hours, people with high socio-economic backgrounds sometimes do choose to eat unhealthy food too. Even though people are well aware of the effect of eating unhealthy foods, it depends on an individual on what they choose to consume.

Based on Fig. 2, unhealthy food and BMI have a significant impact on the depression levels, which seem to directly affect the dependent variables. The prevalence of overweight and obesity among postpartum women in the research sample is noted to be among the highest. From this research, the respondents who eat more unhealthy food and has a high level of BMI are considered to have a high level of depression. Several studies have claimed that depression has a link with maternal obesity [73, 74]. Body dissatisfaction in terms of image, shape, or weight among women would probably affect their mental health.

In the EPDS section, the 10-item questions included anhedonia, self-blame, anxiety, fear or panic, inability to cope, sleeping difficulty, sadness, tearfulness, and self-harm ideas [75]. The descriptive output found that the majority of the respondents suffered from an increased risk of depression levels. The result of depression levels among mothers indeed raised concerns, where they needed help but did not get any. However, in the factor loading analysis, there are three indicators of depression, that have been eliminated from the SEM analysis- Q3, Q5, Q8 (self-blame, fear or panic, and sadness, respectively). Although the descriptive statistics data consider the total score of the EPDS for all 10-items of the depression level measurement, it had decided to remove these indicators, as it had been included in the process of the postpartum depression modeling. The highest loading factor of depression item concerning the anxiety issue (Q4), and the lowest is the anhedonia issue (Q1). For these issues, this research would be an effective platform for medical professionals to keep updated and act towards postpartum women who might feel ashamed or afraid to seek help in preventing them from depression.

Physical activity intervention plays a part in weight loss which happens to be an alternative for the prevention and treatment of the depression symptoms [76]. Moreover, poor sleep incite less motivation to do exercise that leads to weight gain and also obesity-related problemsas well as sleep disturbances [77]. Promotingphysical activity in an individual’s lifestyle can also benefit in averting the potentialenhancement of chronic diseases for which body weight is a risk factor [76]. Consistent with previous literature [78], excessive weight gain probably happen alliance with low physical activity. When ones living with obesity or overweight, their engagement to workout is so frustrating due to discomfort complaints in terms of musculoskeletal and sweating [77]. Prior literature proves that physical activity was correlated with lower BMI and depression levels [79].

Based on Fig. 2, it is observed that the highest coefficient among the variables is the impact of unhealthy food on the depression levels. This corresponded with a previous study by Barker et al. [80], whereby the levels of depression symptoms were linked to unhealthy food consumption [81, 82]. Regarding the research model output, the indirect impact of unhealthy food on the depression levels with the BMI level was identified. Previous studies reported that people who were obese and depressed consumed more unhealthy food [83]. The R-square (R^2^) for the structural model in this research was 0.76. In relative terms, 76% of the variations in depression level were related to socio-demographics, lifestyle, healthy food, unhealthy food, and BMI. Only 24% of the variations correlated with other factors. Thus, it can be concluded that the strength of exogenous and endogenous variables in this research is strong.

However, this research had several limitations, as well. The respondent’s weight and height were self-reported in this study, despite previous research works which have also utilized this method, and although it is valid [84–86], it can be a possible limitation of the study. Furthermore, physical activity that we measured was defined as regular exercise (e.g., fast walking, jogging, cycling, swimming), which were mentioned in the questionnaire. Being a mother had change women’s lifestyle especially to engage in leisure-time especially physical activity [87]. Women seem to be a lack of doing any physical activity because of time constraints and managing their kids [88]. Mothers without husbands or partners were less physically active compared to married mothers [89]. Besides, some of them might work out in different places such as home, gym, park, etc. This indicator is not the main concern in this study. But it is a part of measuring how active the respondents were during the postpartum period.

While the dietary assessment was measured only by using a five-point Likert-type scale. Different BMI category needs different amounts of calories per day. As this research based on self-administrated questionnaires, the Likert-type scale seems to be the easiest way for respondents to report their dietary measurements. Not everyone knows how specific much of the food they consume every day. Yet, we believe that there a lot of ways to measure food intake. For example, the measurement would be in servings [90] or using the MooDFOOD dietary guidelines [91] which been used by recent studies.

Apart from that, the measurement of the depression levels using EPDS was not a substitute for a clinical diagnosis. EPDS was used in this research to determine depression symptoms, which the respondent might face as a form of risk. We acknowledge that many people who suffer from depression did not seek medical help [4]. Medical treatment programs for depression can be effective in reducing depression levels.

**Fig. 3:**
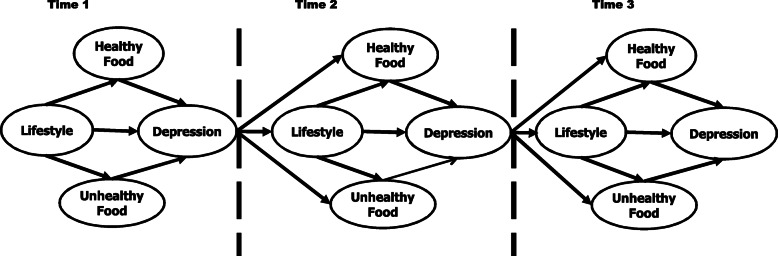
Dynamic SEM framework

In this study, SEM with cross-sectional data could analyze the influence of lifestyle, healthy, and unhealthy food intake on depression. Nevertheless, our research framework, which was presented in Fig. 1, is not capable of studying the vice versa effect of depression on lifestyle, healthy and unhealthy food intake. To overcome this matter, we recommend future studies to apply dynamic SEM with longitudinal data. Figure 3 illustrates an example of dynamic SEM pertaining to our research framework.

The main framework of this study was prepared based on the combination of previous studies in obesity and depression model. However, calorie intake, genetics, and fiber intake are some of the variables that could be obesity indicators that might have been encompassed in our analysis. There were limitations to collect this type of data for this study, and it would have required a different research structure that could not be added in the current research framework. Hence, the analysis of these indicators in future studies is recommended.

## Conclusion

To conclude, this research examined the effects of depression levels in terms of socio-demographics, lifestyle, healthy food, unhealthy food, and BMI. Besides, the hypothesized model in the present study had been indicated as a suitable model for predicting the depression levels among postpartum women. Subsequently, depression levels affect people’s lives (e.g., personal matters, health, eating behavior), and it means clinical intervention is necessary to prevent depression symptoms from exacerbating. This research is the first study on postpartum women diagnosed with depression symptoms, which were carried out using SEM. The factors associated with depression were presented in the theoretical framework. The associated variables and theories were aligned with the Malaysian culture and the associated environment. Thereby, we believe that this research may be advantageous for future works on the postpartum depression modeling, particularly among public health and life science research scholars.

## Data Availability

The data are not publicly available due to the University of Malaya Research Ethics Committee rules and regulations. The data that support the findings of this research are available upon reasonable request from the corresponding author and with permission of the University of Malaya Research Ethics Committee.

## Abbreviations

PPD: Postpartum depression
SEM: Structural Equation Modeling
BMI: Body mass index (BMI)
EPDS: Edinburgh Postnatal Depression Scale
R^2^: R-square
RM: Ringgit Malaysia
CFI: Comparative fit index
NFI: Normed fit index
RFI: Relative fit index
IFI: Incremental fit index
GFI: Goodness of fit index
TLI: Tucker-Lewis index
